# Post-COVID-19 condition is not only a question of persistent symptoms: structured screening including health-related quality of life reveals two separate clusters of post-COVID

**DOI:** 10.1007/s15010-022-01886-9

**Published:** 2022-07-22

**Authors:** Benjamin Giszas, Sabine Trommer, Nane Schüßler, Andrea Rodewald, Bianca Besteher, Jutta Bleidorn, Petra Dickmann, Kathrin Finke, Katrin Katzer, Katja Lehmann-Pohl, Christina Lemhöfer, Mathias W. Pletz, Christian Puta, Stefanie Quickert, Martin Walter, Andreas Stallmach, Philipp Alexander Reuken

**Affiliations:** 1 Department of Internal Medicine IV (Gastroenterology, Hepatology and Infectious Diseases), Jena University Hospital/Friedrich-Schiller-University Jena, Am Klinikum 1, 07747 Jena, Germany; 2 Center for Sepsis Control and Care (CSCC), Jena University Hospital/Friedrich-Schiller-University Jena, Jena, Germany; 3 Public Health Department, City of Jena, 07743 Jena, Germany; 4 Department of Psychiatry, Jena University Hospital/Friedrich-Schiller-University Jena, Jena, Germany; 5 Department of General Practice, University Hospital Jena, Jena, Germany; 6 Department of Anaesthesiology and Intensive Care Medicine, Jena University Hospital/Friedrich-Schiller-University Jena, Jena, Germany; 7 Department of Neurology, Jena University Hospital/Friedrich-Schiller-University Jena, Jena, Germany; 8 Institute of Physiotherapy, Jena University Hospital, Jena, Germany; 9 Institute for Infectious Diseases and Infection Control, Jena University Hospital/Friedrich-Schiller-University Jena, Jena, Germany; 10 Department of Sports Medicine and Health Promotion, Friedrich-Schiller-University Jena, Jena, Germany; 11 Center for Interdisciplinary Prevention of Diseases Related to Professional Activities, Friedrich-Schiller-University Jena, Jena, Germany

**Keywords:** Post-COVID-19, Post-COVID condition, Prevalence, Differentiation, Cluster analysis, QoL

## Abstract

**Purpose:**

Some patients experience long-term sequelae after infection with severe acute respiratory syndrome coronavirus 2 (SARS-CoV-2). However, despite a present post-COVID condition, defined as “any symptom lasting longer than 12 weeks,” only a subset of patients search for medical help and therapy.

**Method:**

We invited all adults with a positive real-time polymerase chain reaction (RT-PCR) for SARS-CoV-2 between March 2020 and September 2021 (*n* = 4091) in the city of Jena to answer a standardized questionnaire including demographic information, the course of the acute infection and current health status. K-means-clustering of quality of life (QoL) was used to explore post-COVID subgroups.

**Results:**

A total of 909 participants at a median interval of 367 (IQR 291/403) days after acute infection were included in the analysis. Of those, 643 (70.7%) complained of having experienced persistent symptoms at the time of the survey. Cluster analysis based on QoL revealed two subgroups of people with persistent post-COVID symptoms. Whereas 189/643 participants (29.4%) showed markedly diminished QoL, normal QoL was detected in 454/643 individuals (70.6%).

**Conclusion:**

Despite persistent symptoms being reported by nearly three quarters of participants, only one-third of these described a significant reduction in QoL (cluster 1), whereas the other two-thirds reported a near-normal QoL (cluster 2), thus indicating a differentiation between “post-COVID disease” and “post-COVID condition”. The prevalence of clinically relevant post-COVID disease was at least 20.7%. Health policies should focus on this subset.

**Supplementary Information:**

The online version contains supplementary material available at 10.1007/s15010-022-01886-9.

## Introduction

Shortly after the first occurrence of severe acute respiratory syndrome 2 (SARS-CoV-2), some patients were found not only to experience acute symptoms, but also to develop long-term sequelae lasting several months or more. If these symptoms persist more than 12 weeks, patients are considered to have a “post-COVID-19 condition” [1]. Overall, more than 50 symptoms have been described as long-term sequelae of SARS-CoV-2-infection, most frequently including chronic fatigue, headache, memory impairment and dyspnea [2]. These data, together with the current rate of infection, suggest that post-COVID will become a major psychosocial and economic challenge. A recent meta-analysis has reported that 80% of all patients develop these long-term sequelae [2]. In contrast, a British study of 4182 patients after SARS-CoV-2-infection has reported symptoms longer than 28 days in only 13.3% of patients and symptoms beyond 12 weeks in only 2.3% of patients [3]. However, data investigating the frequency of post-acute sequelae after SARS-CoV-2 are subject to bias.

One important parameter for identifying patients with a high burden of post-COVID-symptoms is patient-reported quality of life (QoL). The effects of ongoing symptoms on QoL differ depending which symptoms do persist as well as the severity and number of symptoms [4, 5]. Regarding post-COVID-symptoms, the number of patients reporting diminished QoL differs depending on the cohorts and the time after infection; frequencies between 31 and 72% of patients have been reported [6, 7] and partly associated with the COVID-19 disease severity [4, 5].

Therefore, the aim of our study was to analyze the overall burden and effects of post-COVID-symptoms on QoL in a population-based cohort, and to identify phenotypes within the large number of people with persistent symptoms (post-COVID condition) that might benefit from tailored post-COVID treatment.

## Methods

### Study design

All adults registered at the Jena County Public Health Department with RT-PCR-confirmed SARS-CoV-2-infection between March 2020 and September 2021 were eligible to participate in the study voluntarily and anonymously.

Personal letters with invitations, printed questionnaires and a personal access key for online participation were sent between January 14th and 27th, 2022. A total of 4209 adults were invited to participate. Reminders to participate and complete the survey were broadcast via local radio, newspapers and television.

The study was approved by the institutional ethics committee of the Friedrich-Schiller-University-Jena (2021-2454-Bef) and conducted according to the Declaration of Helsinki.

### Measurement tools

In addition to information on sociodemographic status (age, sex, body mass index and previous illnesses), we asked participants about the course of acute SARS-CoV-2-infection, including the modified WHO 8-point-ordinal-scale [8] and SARS-CoV-2 vaccination-status before infection and at the time of the survey. Similarly, our survey collected detailed data on post-infectious symptoms and current satisfaction with health.

With the survey instruments commonly used in our post-COVID outpatient clinic at Jena-University-Hospital [9, 10], we used globally established and validated questionnaires including the Short-Form-36 Health-Survey (SF-36V2), Fatigue-Assessment-Scale (FAS [11]) and Patient-Health-Questionnaire-9 (PHQ-9 [12]). All questionnaires and tests were delivered to participants in German language only and were interpreted according to the current manuals.

### Neuropsychiatric assessment

Participants were considered to have fatigue or severe fatigue if they had ≥ 22 points or ≥ 35 points on the FAS questionnaire [11], respectively. Depression was defined as absent if patients scored ≤ 4 points on the PHQ-9, mild if patients scored 5–9 points, moderate if patients scored 10–14 points, and severe if patients scored ≥ 15 points [12].

### Quality of life

The SF-36 assesses physical and mental QoL by evaluating health status in eight dimensions: physical functioning, role-physical, bodily pain, general health, vitality, social functioning, role-emotional, mental health. Summarizing the QoL, physical and mental component sum scores were determined according to the official assessment tool [13]. To assess the QoL, we compared the results with those in the general population and already diagnosed post-COVID patients. The control data for the representative comparison with the German normal population were taken from the German-Health-Survey-and-Examination-for-Adults (DEGS1, *n* = 8152) [14], including adults 18–79 years of age.

Patients who presented to the post-COVID outpatient clinic of Jena University Hospital (as already described in detail [9]) from March 2021 onward with diagnosed post-COVID, fully answered the SF-36V2 and were living outside Jena were included (*n* = 431, partly already published [15]), whereas patients residing in Jena were excluded to avoid duplication between the study and positive control group.

### Statistics

For descriptive analysis, we summarized participant characteristics as absolute and relative frequencies for categorical variables, and as medians with first and third quartiles for numeric variables.

For analysis of differences between groups, Pearson's chi-square-test and Fisher's exact-test were used for categorical variables, whereas the Mann–Whitney-*U*-test and Kruskal–Wallis-test were used for numeric variables. A significance level of *α* < 0.05 (two-sided) was used. For comparisons of more than two groups, we adjusted the results with Bonferroni–Holm post hoc-test.

To identify the clinically important differences between two groups in SF-36V2 dimension and sum-scales, we used Mann–Whitney-*U*-test and effect size *r*. A significant difference was assumed only if a significant difference was observed with at least an intermediate effect size *r* ≥ 0.3.

Non-hypothesis-driven *k*-means clustering accounted for numerical data of all dimensions, and the sum scores of SF-36V2 were used to study the underlying clinical patterns of post-COVID. To identify the best possible number of clusters, the within-cluster sum of squared error was plotted between 1 and 10 clusters (Supplementary Fig. 1). With the elbow-method, two clusters were selected.

Univariable and multivariable logistic regression models were used to identify and evaluate possible predictors of post-COVID disease. The multivariable models were adjusted for sex and age accordingly. We report (adjusted) odds ratios (ORs) with 95% confidence interval (CIs) and two-sided *p* values. All analyses were performed in IBM SPSS version 26 and GraphPad Prism6.

## Results

### Study population

**Fig. 1:**
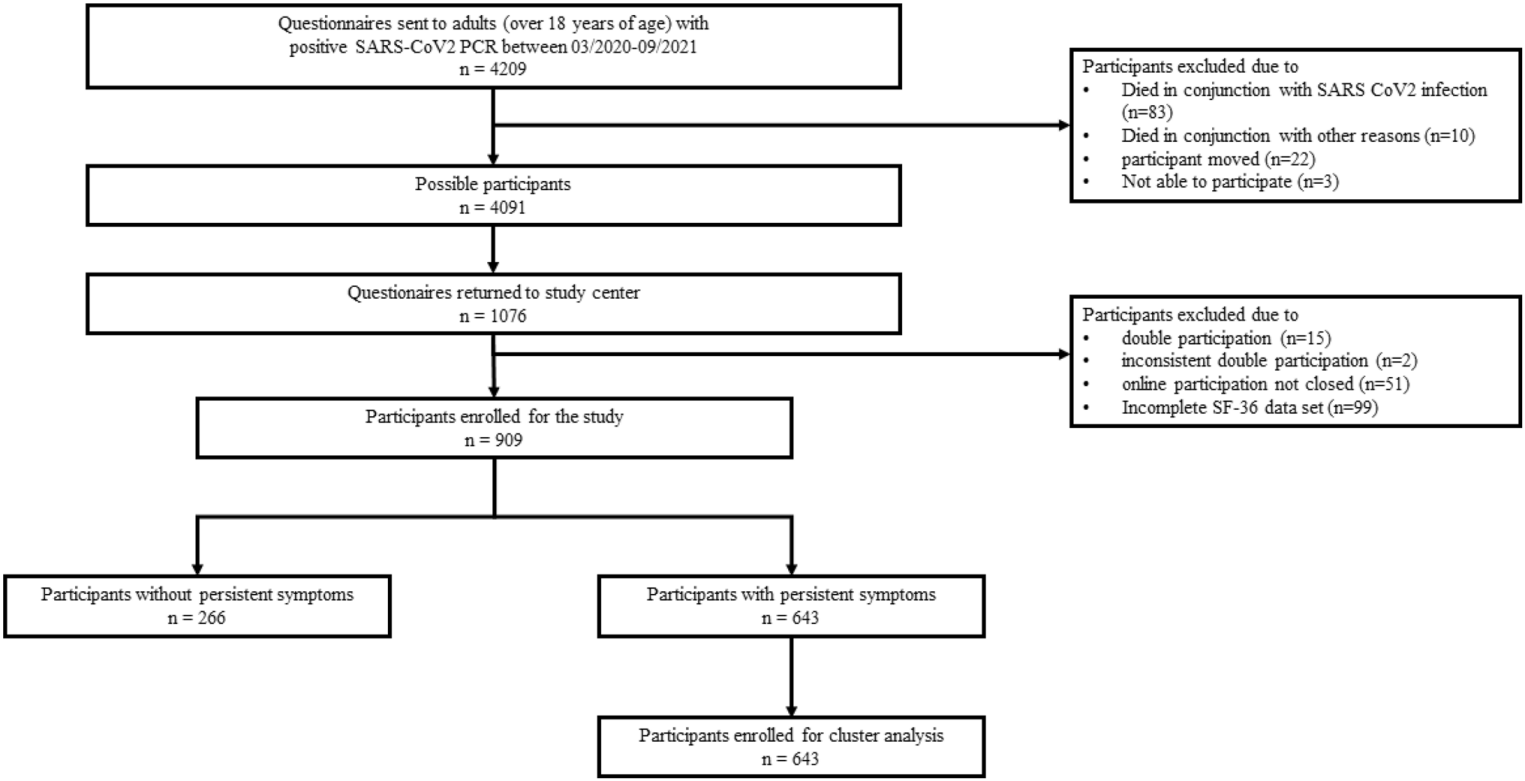
Participant flow of the survey

We invited 4209 individuals to answer the questionnaire. A total of 118 participants were knowingly unable to participate in the study (93 people died between infection and sending of the questionnaire, 22 questionnaires could not be delivered because of incorrect postal addresses, and three people were unable to participate because of existing mental illnesses). In total, 1009 (24.0%) people answered the questionnaire. We excluded 99 people from our analyses because of incomplete SF-36 responses. The final study population included 909 of 4091 (22.2%) participants a median of 367 (IQR 291/403) days after SARS-CoV-2-infection (Fig. 1).

The median age of the study population was 47 (IQR 33/59) years; 530 (59.3%) were women, and the acute infection occurred more than nine months prior in 822 participants (90.7%). Inpatient treatment was necessary in 33 individuals (3.7%). A total of 101 people (10.3%) had been vaccinated at least once before SARS-CoV-2-infection. In contrast, 770 (88.2%) of all participants and 704 (89.7%) of all non-vaccinated individuals reported a vaccination in the interval after infection (Table 1).

**Table 1:** Absolute (*n*) and relative frequencies (%), or medians together with first and third quartiles (Q1, Q3) from the total cohort, and post-COVID disease and condition cluster are provided

Characteristics	Number of participants with missing information	Total cohort (*n* = 909)	Number of participants with missing information	Post-COVID disease cluster 1 (*n* = 189)	Number of participants with missing information	Post-COVID condition cluster 2 (*n* = 454)	*p* value
Digital participation *n* (%)		357 (39.3%)		69 (36.5%)		151 (33.3%)	0.429
Female sex; *n* (%)	15	530 (59.3%)	2	132 (70.6%)	2	272 (60.2%)	0.013
Age, in years; median (Q1/Q3), minimum–maximum	12	47 (33/59) 18–95	2	53 (41/62) 18–95	1	48 (35/60) 18–91	0.029
18–40		313 (34.9%)		38 (20.3%)		154 (34%)	
40–65		475 (53%)		118 (63.1%)		237 (52.3%)	
> 65		109 (12.2%)		31 (16.6%)		62 (13.7%)	
Body mass index (BMI) median (Q1/Q3), minimum–maximum	19	24.7 (22.1/28.5) 16.7–57.8	4	27.3 (23.4/30.3) 16.7–57.4	3	24.5 (22.1/28.1) 17–46.1	< 0.001
Underweight		20 (2.2%)		3 (1.6%)		8 (1.8%)	
Normal weight		448 (50.3%)		67 (36.2%)		237 (52.5%)	
Pre-obesity		264 (29.7%)		64 (34.6%)		127 (28.2%)	
Obesity grade 1		126 (14.2%)		37 (20%)		67 (14.9%)	
Obesity grade 2		22 (2.5%)		8 (4.3%)		10 (2.2%)	
Obesity grade 3		10 (1.1%)		6 (3.2%)		2 (0.4%)	
Out-patient only; *n* (%)	8	868 (96.3%)	1	181 (96.3%)		430 (94.7%)	
In-patient; *n* (%)	8	33 (3.7%)	1	7 (3.7%)		24 (5.3%)	
Needing oxygen support; *n* (%)	8	17 (1.9%)	1	2 (1.1%)		14 (3.1%)	
ICU stay; *n* (%)	8	9 (1.0%)	1	1 (0.5%)		8 (1.8%)	
invasive ventilation; n (%)	8	3 (0.3%)	1	0 (0%)		3 (0.7%)	
WHO grade	8		1				< 0.001
1; *n* (%)		246 (27.3%)		20 (10.6%)		116 (25.6%)	
2; *n* (%)		622 (69%)		161 (85.6%)		314 (69.2%)	
3; *n* (%)		16 (1.8%)		5 (2.7%)		10 (2.2%)	
4; *n* (%)		8 (0.9%)		1 (0.5%)		6 (1.3%)	
5; *n* (%)		6 (0.7%)		1 (0.5%)		5 (1.1%)	
6; *n* (%)		3 (0.3%)		0 (0%)		3 (0.7%)	
7; *n* (%)				0 (0%)		0 (0%)	
Vaccination status	18		5		3		0.715
No		802 (90.0%)		165 (89.7%)		408 (90.5%)	
Incomplete		44 (4.9%)		8 (4.3%)		25 (5.5%)	
Complete (min. 2)		45 (5.1%)		11 (6.0%)		18 (4.0%)	
Vaccination after infection	36	770 (88.2%)	10	153 (85.5%)	11	405 (91.4%)	0.027
Time after infection	3		1		1		0.697
≥ 3 months		47 (5.2%)		10 (5.3%)		19 (4.2%)	
≥ 6 months		37 (4.1%)		9 (4.8%)		16 (3.5%)	
≥ 9 months		229 (25.3%)		44 (23.4%)		126 (27.8%)	
≥ 12 months		450 (49.7%)		93 (49.5%)		224 (49.4%)	
≥ 15 months		93 (10.3%)		23 (12.2%)		43 (9.5%)	
≥ 18 months		50 (5.5%)		9 (4.8%)		25 (5.5%)	
Days after positive testing median (Q1/Q3)	39	367 (291/403)	9	368 (290.25/404.75)	20	359.59 (292.75/404)	0.566
No preexisting conditions	11	469 (52.2%)	1	66 (35.1%)	1	235 (51.9%)	< 0.001
Obesity		158 (17.4%)		51 (27.0%)		79 (17.4%)	0.006
Hypertension	11	213 (23.7%)	1	65 (34.6%)	1	109 (24.1%)	0.006
Diabetes mellitus	11	45 (5.0%)	1	23 (12.2%)	1	13 (2.9%)	< 0.001
Dialysis	11	2 (0.2%)	1	1 (0.5%)	1	0 (0%)	
Kidney disease	11	14 (1.6%)	1	4 (2.1%)	1	8 (1.8%)	0.759
Liver disease	11	32 (3.6%)	1	14 (7.4%)	1	12 (2.6%)	0.005
Lung disease	11	94 (10.5%)	1	39 (20.7%)	1	43 (9.5%)	< 0.001
Cancer	11	30 (3.3)	1	13 (6.9%)	1	12 (2.6%)	0.011
Psychologic disorder	11	14 (1.6%)	1	6 (3.2%)	1	5 (1.1%)	0.006
Active smoking	19	124 (13.9%)	3	33 (17.7%)	5	53 (11.8%)	0.05
Active and former smoking	19	207 (23.2%)	3	55 (29.5%)	5	94 (20.9%)	0.02
Medication	14	404 (45.1%)	2	128 (68.4%)	2	202 (44.5%)	< 0.001
Polypharmacy	14	42 (4.7%)	2	25 (13.4%)	2	15 (3.3%)	< 0.001

Relative frequencies are associated with participants who provided information on the specific characteristics

### Prevalence of persistent symptoms (post-COVID condition) after SARS-CoV-2-infection

A total of 643 (70.7%) of the 909 participants reported having persistent symptoms at the time of the interview. Of those, 110 participants (17.1%) reported one symptom, and 533 (82.9%) reported two or more symptoms (Supplementary Fig. 2). Depending on the time interval between acute infection and answering the questionnaire, the proportion of participants with persistent symptoms ranged between 61.7 and 74.2% (Supplementary Fig. 3). The absolute number of individuals in each 3-month period varied from 37 to 450 participants; however, this large range corresponds to the number of overall infections in the related period (Supplementary Fig. 4). Fatigue [364/876 (41.6%)], sleep disturbance [357/879 (40.6%)], pain [317/849 (37.3%)], memory impairment [243/872 (27.9%)], and respiratory problems [230/867 (26.5%)] were most frequently reported (Supplementary Fig. 5).

### Quality of life after survival from SARS-CoV-2-infection

**Fig. 2:**
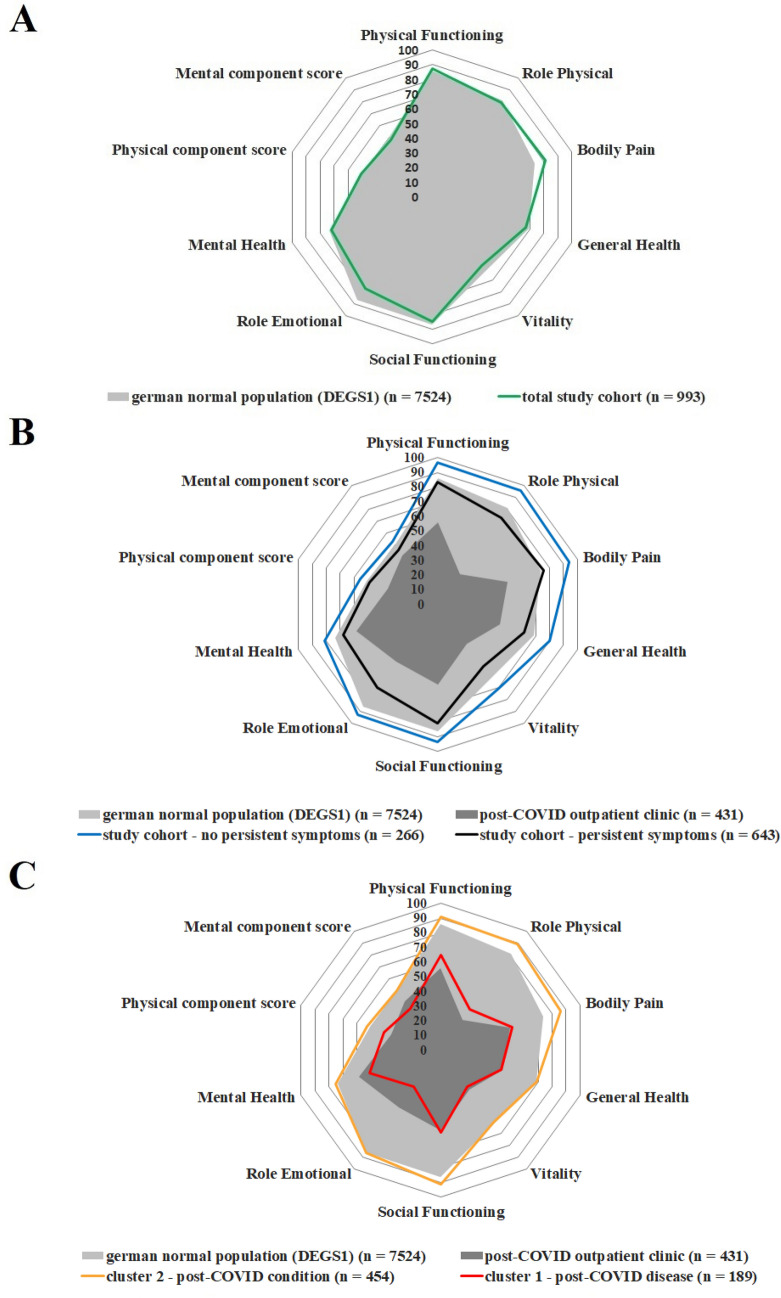
Results of SF-36 itemized by dimensions and component summary scores. **A** Comparison of the total study group to the German normal data from Study on the Health of Adults in Germany–Wave 1 (DEGS1; *n* = 7524). **B** Analysis of the cohort according to persistent symptoms. Both resulting groups are compared with German normal population (DEGS1; *n* = 7524) and patients with diagnosed post-COVID from our specialized outpatient clinic (*n* = 431). **C** Clustering of the group with persistent symptoms according to the SF-36 scores. This clustering indicated two groups: one with significantly diminished QoL (post-COVID disease; *n* = 189) and a second with near-normal QoL (post-COVID condition; *n* = 454)

The physical and mental component sum scores [51.0 (SD 9.0) in total cohort vs. 50.8 (SD 9.3] in DEGS1 (*p* = 0.82) and 47.95 (SD 10.4) in total cohort vs. 49.9 (SD 9.79) in DEGS1 (*p* < 0.001, *r* = 0.06)) indicated that the QoL of the analyzed total cohort was largely consistent with those in the German normal population (Fig. 2A). Closer analysis revealed that only the dimensions of bodily pain [81.1 (SD 25.3) vs. 73.7 (SD 26.5), *p* < 0.001, *r* = 0.08] and role emotional [76.8 (SD 34.7) vs. 86.4 (SD 21.0), *p* < 0.001, *r* = 0.03] showed differences above five points.

To analyze the burden of post-COVID, we divided the study population into participants with [643/909 (70.7%), previously defined as post-COVID condition] and without [266/909 (29.3%)] persistent complaints. A significant and relevant decrease in QoL in all dimensions and sum-scores (*p* < 0.001, minimum *r* = 0.31) except the social functioning dimension (*p* < 0.001, *r* = 0.27) was observed. Each of the post-COVID-associated symptoms had a different effect on the dimensions of QoL (Supplementary Table 1).

To further assess the effects of post-COVID on QoL, we compared the participants with persistent symptoms to a cohort of 431 already diagnosed post-COVID patients presenting to our post-COVID outpatient clinic. However, the groups markedly and clinically significantly differed in most dimensions and sum scores [*p* < 0.001, minimum *r* = 0.4, except role emotional (*p* < 0.001, *r* = 0.25), mental health (*p* < 0.001, *r* = 0.24) and mental component sum score (*p* < 0.001, *r* = 0.22)] (Fig. 2B).

Given the large differences, a more specific cluster analysis was performed. According to the individual components of the SF-36, two clusters were identified (Fig. 2C; Supplementary Figs. 1 and 6).

### Characteristics of the identified post-COVID clusters

Among the 643 post-COVID participants, 189 (29.4%) were classified in cluster 1, and 454 (70.6%) were assigned in cluster 2. Cluster 1 was characterized by a significantly diminished self-reported and measured QoL and greater dissatisfaction with their own health, and therefore was denoted as the post-COVID disease cohort (Fig. 2C; Supplementary Fig. 7).

People within the post-COVID disease cohort were older [median (Q1/Q3) of 53 (41/62) vs. 48 (35/60), *p* = 0.029], significantly more likely to be women (70.6% vs. 60.2%, *p* = 0.013) and more likely to have a comorbidity (64.9% vs. 42.1%, *p* < 0.001; Table 1).

Comparing these cohorts with the already diagnosed post-COVID patients from the outpatient clinic of Jena-University-Hospital, we observed substantial similarities to cluster 1 (maximum *r* = 0.22) and relevant differences with respect to cluster 2 (minimum *r* = 0.41; Fig. 2C). Although significant differences were also found in comparison to cluster 1, given the group sizes, the effect sizes were all small.

**Fig. 3:**
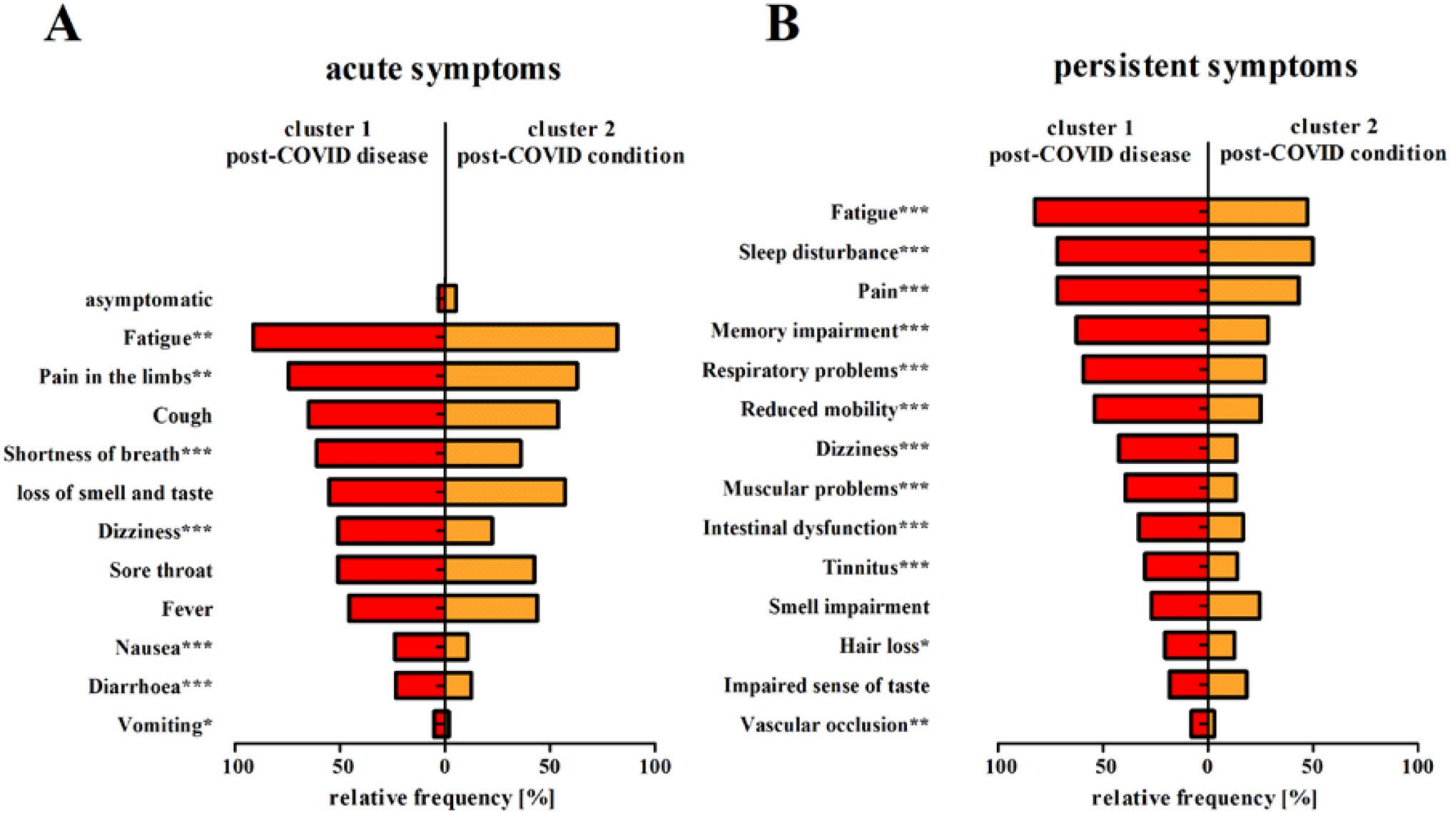
Frequency of acute (**A**) and persistent (**B**) post-COVID-associated symptoms according to cluster classification. The proportions of patients with each symptom are shown (**A**: 188 participants in cluster 1 and 453 participants in cluster 2, **B**: fatigue (187/444), sleep disturbance (188/445), pain (181/431), memory impairment (187/441), respiratory problems (187/439), reduced mobility (183/441), dizziness (181/434), muscular problems (180/433), intestinal dysfunction (182/441), tinnitus (180/435), smell impairment (182/439), hair loss (181/442), impaired sense of taste (180/440), vascular occlusion (174/437); *p ≤ 0.05; **p ≤ 0.01; ***p ≤ 0.001)

**Fig. 4:**
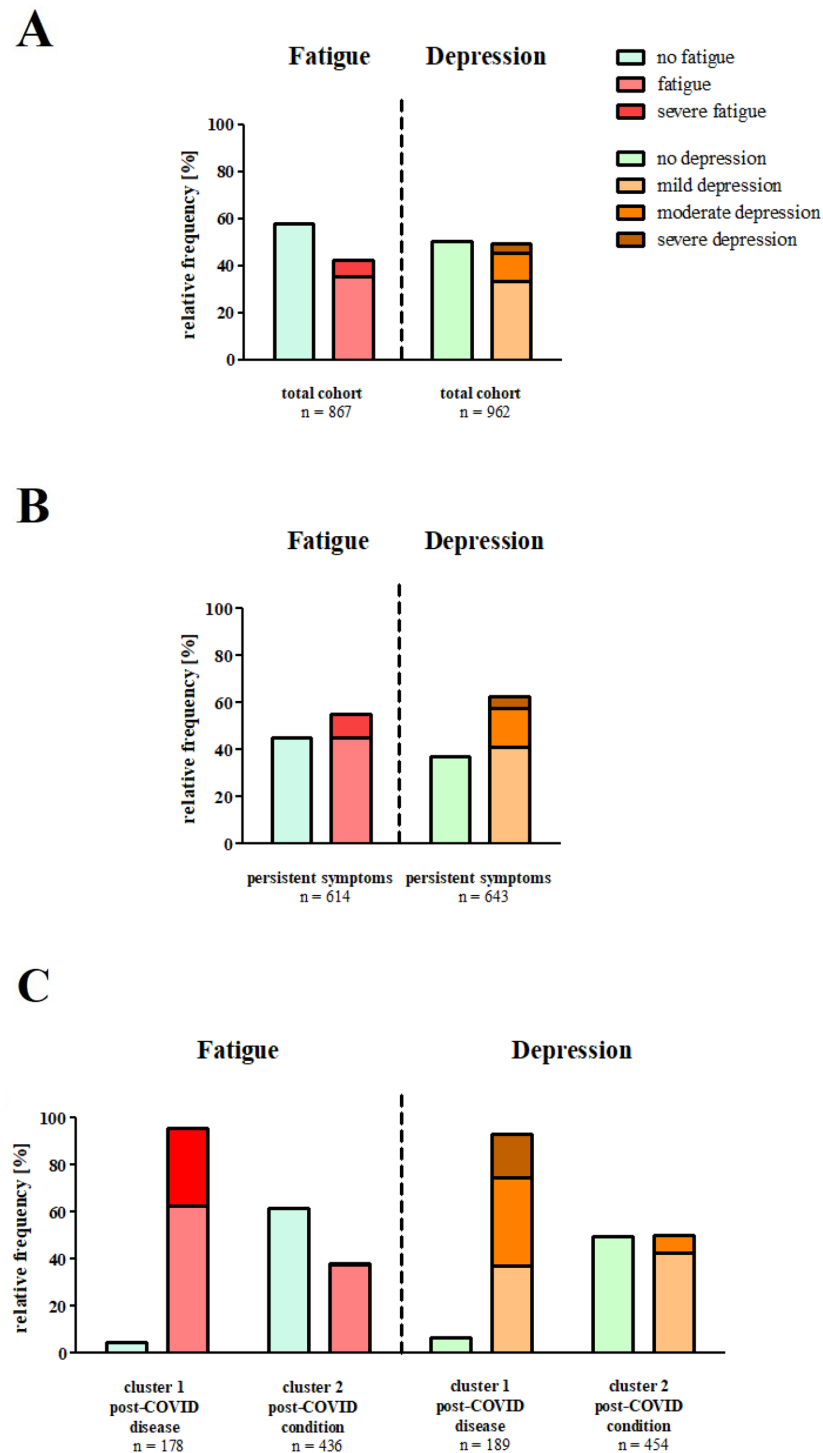
Relative frequencies of fatigue and depression according to the FAS and PHQ-9 questionnaires in the total cohort (**A**) and subgroups according to persistent symptoms (**B**) and post-COVID cluster classification (**C**)

More than half the individuals in post-COVID disease cohort reported experiencing fatigue [82.4% (154/187), vs. 47.3% (210/444) in cluster 2, *p* < 0.001], sleep disturbances [71.8% (135/188), vs. 49.9% (222/445) in cluster 2, *p* < 0.001], pain [71.8%, vs. 43.4% in cluster 2, *p* < 0.001], memory impairments [62.6% (117/187), vs. 28.6% (126/441) in cluster 2, *p* < 0.001], respiratory problems [59.4% 6(111/187), vs. 27.1% (119/439), *p* < 0.001] and reduced mobility [54.1% (99/183), vs. 25.2% (111/441) in cluster 2, *p* < 0.001; Fig. 4]. In addition, the number of persistent symptoms differed between the identified clusters [median (Q1/Q3) of 6 (4/8), vs. 3 (2/4.25) in cluster 2, *p* < 0.001; Fig. 3; Table 1].

However, even with acute infection, participants with post-COVID disease experienced more symptoms on average [median (Q1/Q3) of 6 (4/7) vs. 4 (3/6) in cluster 2, *p* < 0.001; Fig. 3; Supplementary Fig. 8].

### Prevalence of depression and fatigue among SARS-CoV-2-survivors

Screening for psychiatric complaints with a structured questionnaire-based assessment in the total cohort revealed evidence of fatigue in 368 individuals (42.5% of 867 who completed the FAS questionnaire) and signs of depression in 450 individuals (49.5% of all participants; Fig. 4A). In subgroup analysis of all people with persistent symptoms, the number of participants with fatigue [54.9% (337/614 participants) vs. 12.3% (31/253 participants), *p* < 0.001] and depression [66.8% (404/643 participants) vs. 17.3% (46/266 participants), *p* < 0.001] increased (Fig. 4B).

Analysis of the identified post-COVID-clusters revealed a significantly higher proportion of affected individuals with fatigue [95.5% (170/178 participants) vs. 38.3% (167/436 participants) in cluster 2, *p* < 0.001] and depression [93.1% (176/189 participants) vs. 50.2% (228/454 participants) in cluster 2, *p* < 0.001] in cluster 1 (Fig. 4C).

Furthermore, a markedly greater co-occurrence of fatigue and depression was observed in cluster 1 than cluster 2 [169/178 participants (91.4%) in vs. 138/454 (30.9%) in cluster 2; Supplementary Fig. 9].

### Predictors for the development of post-COVID disease

An unadjusted analysis predicted differences in age and sex: older and female participants had a higher risk of being in the post-COVID disease suspected group. Age- and sex-stratified multivariate binary- regression identified several pre-existing diseases as predictors of post-COVID-disease, including diabetes mellitus [OR 3.93 (95% CI 2.044, 7.556)), *p* < 0.001], chronic liver and lung diseases [OR 2.986 (95% CI 1.422, 6.271), *p* = 0.004, OR 2.951 (95% CI 1.868, 4.661), *p* < 0.001], obesity [OR 1.956 (95% CI 1.319, 2.899), *p* < 0.001] and arterial hypertension [OR 1.727 (95% CI 1.148, 2.598), *p* = 0.009]. Furthermore, development of a post-COVID-disease was more likely in participants with polypharmacy [OR 5.036 (95% CI 2.506, 10.121), *p* < 0.001]. Initially more severe infection [OR 1.539 (1.198, 1.976), *p* < 0.001] was also associated with post-COVID-disease, and the occurrence of certain symptoms in the acute infection phase as well as the post-infectious phase were independent risk factors (Supplementary Tables 2, 3). In contrast, the absence of previous illnesses could be characterized as protective [OR 0.464 (95% CI 0.322, 0.669), *p* < 0.001].

However, inpatient treatment [OR 1.012 (95% CI 0.423, 2.421), *p* = 0.978], the time interval between the acute infection and the survey [OR 0.999 (95% CI 0.855, 1.168), *p* = 0.993], and the need for oxygen supply [OR 0.291 (95% CI 0.017, 4.909), *p* = 0.392] or ICU treatment [OR 0.138 (95% CI 0.003, 5.951), *p* = 0.302] had no significant influence on the development of post-COVID-disease (Supplementary Table 4).

## Discussion

The medical and societal challenge of the COVID-19-pandemic is omnipresent, with persistent symptoms that continue to plague some COVID-19-survivors for months. Given the high incidence of infections worldwide, affecting half a billion people, we anticipate a medical and societal threat from post-COVID by extended absences due to illness and a potentially globally reduced workforce even after the pandemic is contained. In the group of participants with a defined post-COVID condition, only one-third reported significantly diminished QoL (cluster 1), whereas the remainder reported near-normal QoL (cluster 2), thus indicating the need for further differentiation of patients into a QoL-reducing post-COVID disease and a post-COVID condition with preserved QoL.

Our closed population-based survey of all 4209 infected adult residents in the city of Jena (approximately 111,000 inhabitants) is one of the largest surveys of an unselected population on the topic of post-COVID worldwide, with 909 participants and an evaluable response rate of 21.6% [16, 17].

Overall, the data from our study show that approximately 70.7% of people continually experience at least one symptom after SARS-CoV-2-infection. In agreement with previously published work on post-COVID, SARS-CoV-2-survivors complained primarily of fatigue, sleep disturbance, pain and memory impairment [18, 19].

A question remaining to be clarified is whether the chronically symptomatic individuals actually show a substantial decrease in their QoL and thus potentially seek access to the health care system more often.

We demonstrated that the QoL in the entire cohort differed only marginally from the normal population, thus indicating that a substantial proportion of SARS-CoV-2-infections heal without long-term sequelae, in agreement with the results of several other studies [3, 18–20]. We observed only a slight decrease in the role emotional dimension in the participants, possibly as a result of the general measures in managing the COVID-19 pandemic [21]. This finding is in line with the results of several other studies reporting a decline in QoL among COVID-19-survivors, depending on sociodemographic factors and belief in the negative effects of recent SARS-CoV-2-infection [22], which has also been observed in family members of ICU COVID-19-survivors [23].

Despite the good overall QoL in our study, through cluster analysis, we identified significantly different subgroups among the participants with persistent symptoms. Although most participants with persistent symptoms reported near-normal QoL, one-third of participants had poor self-reported QoL. Interestingly, in all dimensions of the SF-36, this cluster had high similarity to our patients from the Jena-University-Hospital post-COVID outpatient clinic, thus highlighting that this group is more likely to seek medical help [9, 15, 24]. This result suggests that the combination of persistent symptoms and poor self-reported health condition may identify patients with significant, clinically relevant problems requiring further treatment.

We found a significantly higher number of comorbidities in the identified post-COVID-disease cohort (cluster 1) than the post-COVID condition cluster 2, in agreement with other previously published studies [3, 25]. Equivalently, polypharmacy (defined as a minimum of five daily medications) was also identified as a crucial risk factor. The identification of comorbidities is of special interest, because the current concepts define post-COVID as “persistent symptoms that cannot be explained by other conditions” [26]. As described by other groups, owing to the increased risk, pre-diseased patients must be closely monitored for early detection of post-COVID-disease [25].

Another identified risk factor was the initial severity of the infection. This finding is consistent with those from several other studies [20, 27], whereas other studies have not reported this association [9]. A Delphi consensus of WHO-network has described the constellation of symptoms that occur months after a SARS-CoV-2-infection as a “post-COVID-19 condition” [1]. In general, disease is defined as a disturbance of normal physical or mental functions that reaches a degree with a perceived negative effect on the performance and well-being of a living being, either subjectively or objectively. Given the sometimes-substantial impairments of post-COVID patients, the term "disease" should be considered for the description of post-COVID condition.

### Strengths and limitations

The present study is one of the largest population-based cross-sectional surveys associated with post-COVID. It prevents preselection of specific criteria such as constellations of symptoms and initial disease severity, thus clearly distinguishing this study from previous research work [9, 18, 28].

A major limitation is the lack of a control group. However, the QoL of the total cohort corresponds to the normal German population [14]; therefore, both the already published normal population and the participants without persistent complaints served as a separate control group.

Nevertheless, the ability to evaluate the symptoms regarding SARS-CoV-2-specificity was limited in this study. All data were based on self-reporting by the patients and therefore might have been biased: affected people were asked to remember their symptoms retrospectively, and affected patients might have been more likely to participate in the study. Additionally, questionnaires were only assessed in German language and sent via postal services which could lead to reduced participation. Given that more than three-quarters of people (78.4%, *n* = 3300) infected with SARS-CoV-2 did not respond to the questionnaire, and assuming conservatively that those people were symptom-free, the proportion of clinically relevant patients with post-COVID-disease was reduced to a maximum of 4.5% (189 of 4209 individuals tested positive for SARS-CoV-2). This finding is in line with analyses by the German health insurance companies, in which medical care has been reported to be used in 6% of SARS-CoV-2-survivors [29]. Finally, we included patients infected before October 2021 to allow all patients a complete 12-week-period after the infection; therefore, we cannot draw conclusions regarding the effects of certain viral variants being more likely to cause more (delta) or less (omicron) severe courses in the acute phase.

## Conclusion

In summary, nearly three-quarters of SARS-CoV-2-survivors experienced persistent symptoms, whereas only one-third had significantly diminished QoL, thus highlighting QoL as an additional main criterion for the assessment of post-COVID condition.

Furthermore, our study detected a prevalence of 20.8% of relevant post-COVID disease among SARS-CoV-2-survivors, a proportion higher than the prevalence of persistent symptoms after other viral infections [30].

Therefore, the combination of persistent symptoms and diminished QoL should be defined as “post-COVID disease” to specify the group of patients who should be focused on in intervention trials and health policies.

## Supplementary Information

Below is the link to the electronic supplementary material.

Supplementary file1 (PDF 628 kb)
