# Supplementary material for: Post-COVID-19 condition is not only a question of persistent symptoms: structured screening including health-related quality of life reveals two separate clusters of post-COVID
Source: Infection. 2022 Jul 22;51(2):365–77. doi: 10.1007/s15010-022-01886-9 (PMC9307219; doi:10.1007/s15010-022-01886-9)

Table of content.

Supplementary Table 1. Results of multivariable logistic regression modeling to assess the influence of persistent symptoms on the dimensions and sum scores of SF-36 in all participants. Odds ratios and 95% CIs are shown (p value in all cases <0.05). 2

Supplementary Table 2. Results of uni- and multivariable binary logistic regression modeling to assess the risk of specific persistent symptoms in the occurrence of post-COVID-disease in all participants with sustained complaints. 3

Supplementary Table 3. Results of uni- and multivariable binary logistic regression modeling to assess the risk of symptoms within the acute phase of infection for the occurrence of post-COVID-disease in all participants. .. 3

Supplementary Table 4. Results of uni- and multivariable binary logistic regression modeling to assess risk factors for the occurrence of post-COVID-disease in the total cohort (n=909). 4

Legend of the supplementary figures 5

Supplementary Figure 1. Within-cluster sum of square (WCSS) depending on the number of clusters in k-means analysis of the cohort with persistent symptoms (n=643). 6

Supplementary Figure 2. Number of symptoms of acute SARS-CoV-2 infection and persistent symptoms in a population-based cohort of SARS-CoV-2 survivors (maximum of acute phase=10, prolonged and persistent phase=14). 6

Supplementary Figure 3. Time-dependent distribution of participants. A. Median time interval (days) between acute infection and the survey. Time-dependent representation of the frequency of participants with persistent complaints (B) and the identified post-COVID clusters (C) related to the total cohort. 7

Supplementary Figure 4. Comparison of the monthly incidence in Jena with the number of survey participants infected with SARS-CoV-2 in the given month. 7

Supplementary Figure 5. Frequency of persistent post-COVID associated symptoms in a population-based cohort of SARS CoV-2 survivors. The proportions of patients with each symptom are shown. 8

Supplementary Figure 6. Analysis of the individual properties integrated in the cluster analysis (SF-36 dimensions and sum scales), plotted on a normalized scale (z-score) (* p<0.001). 9

Supplementary Figure 7. Relative frequency of satisfaction with personal health (A) and self-reported QoL (B) on a Likert scale in SARS-CoV-2 survivors, according to the occurrence of persistent symptoms and the influence of SF-36 results. The proportions of participants are shown as diverging stacked bar charts (* p<0.001 Kruskal-Wallis test). 10

Supplementary Figure 8. Frequency of acute symptoms in a population-based cohort of SARS CoV-2 survivors, according to persistent symptoms. The proportions of participants with each symptom as well as the proportion of asymptomatic individuals are shown (* p≤0.05; ** p≤0.01; *** p≤0.001). 11

Supplementary Figure 9. Venn diagram of identified fatigue and depression in the post-COVID cohort and specified post-COVID disease cluster, according to FAS and PHQ-9. Overall, 307 people (48.6%) with persistent symptoms, and 169 (91.4%) and 138 (30.9%) in the identified post-COVID subclusters, respectively, experienced both fatigue and depression. The proportion of co-occurrence of having both, fatigue and depression was significantly higher in post-COVID disease cluster 1 12

Supplementary Table 1. Results of multivariable logistic regression modeling to assess the influence of persistent symptoms on the dimensions and sum scores of SF-36 in all participants. Odds ratios and 95% CIs are shown (p value in all cases <0.05).

	Number of symptoms	Fatigue	Pain	Sleep disturbance	Memory impairment
**Physical functioning**	-3.214 (-3.561, -2.868)	-12.489 (-14.72, -10.258)	-12.164 (-14.635, -9.693)	-8.398 (-10.75, -6.046)	-12.821 (-15.267, -10.376)
**Role physical**	-5.677 (-6.316, -5.038)	-26.397 (-30.329, -22.466)	-24.388 (-28.822, -19.953)	-19.057 (-23.265, -14.849)	-26.818 (-31.184, -22.452)
**Bodily pain**	-4.489 (-4.984, -3.994)	-17.652 (-20.811, -14.492)	-26.26 (-29.481, -23.039)	-13.864 (-17.163, -10.564)	-17.107 (-20.6, -13.615)
**General health**	-3.573 (-3.945, -3.202)	-17.108 (-19.397, -14.819)	-15.3 (-17.908, -12.693)	-12.877 (-15.342, -10.412)	-15.314 (-17.92, -12.708)
**Vitality**	-3.966 (-4.38, -3.551)	-22.546 (-24.964, -20.127)	-15.679 (-18.589, -12.769)	-16.322 (-19.014, -13.629)	-19.027 (-21.861, -16.194)
**Social functioning**	-3.52 (-4.018, -3.022)	-15.94 (-18.99, -12.891)	-12.714 (-16.19, -9.237)	-11.676 (-14.876, -8.475)	-15.664 (-19.048, -12.281)
**Role emotional**	-5.435 (-6.159, -4.711)	-25.395 (-29.789, -21.0)	-19.723 (-24.683, -14.763)	-20.902 (-25.501, -16.303)	-30.648 (-35.359, -25.936)
**Mental health**	-2.836 (-3.205, -2.467)	-14.5 (-16.722, -12.279)	-10.532 (-13.096, -7.967)	-12.044 (-14.381, -9.708)	-13.543 (-16.024, -11.061)
**Physical component score**	-1.613 (-1.775, -1.451)	-6.562 (-7.603, -5.521)	-7.961 (-9.076, -6.846)	-4.496 (-5.606, -3.387)	-6.136 (-7.302, -4.97)
**Mental component score**	-1.606 (-1.822, -1.39)	-8.626 (-9.913, -7.338)	-5.412 (-6.917, -3.908)	-7.027 (-8.388, -5.665)	-8.616 (-10.043, -7.188)

	Respiratory problems	Dizziness	Reduced mobility	Muscular problems	Intestinal dysfunction
**Physical functioning**	-16.819 (-19.245, -14.393)	-16.477 (-19.507, -13.447)	-16.985 (-19.707, -14.262)	-17.145 (-20.237, -14.054)	-12.2 (-15.31, -9.09)
**Role physical**	-26.169 (-30.722, -21.616)	-26.072 (-31.655, -20.489)	-26.751 (-31.772, -21.729)	-27.718 (-33.397, -22.04)	-16.188 (-21.892, -10.484)
**Bodily pain**	-19.974 (-23.516, -16.431)	-18.133 (-22.514, -13.752)	-28.631 (-32.334, -24.928)	-22.656 (-27.035, -18.277)	-16.832 (-21.243, -12.421)
**General health**	-17.486 (-20.105, -14.866)	-16.251 (-19.542, -12.959)	-16.387 (-19.356, -13.418)	-14.854 (-18.257, -11.451)	-12.211 (-15.563, -8.859)
**Vitality**	-15.789 (-18.806, -12.772)	-17.144 (-20.82, -13.468)	-14.563 (-17.956, -11.169)	-17.543 (-21.314, -13.771)	-13.979 (-17.706, -10252)
**Social functioning**	-12.625 (-16.176, -9.075)	-17.077 (-21.317, -12.837)	-13.479 (-17.375, -9.582)	-18.491 (-22.82, -14.162)	-16.229 (-20.443, -12.016)
**Role emotional**	-20.989 (-26.121, -15.857)	-26.437 (-32.552, -20.323)	-22.624 (-28.244, -17.004)	-29.819 (-36.038, -23.601)	-19.406 (-25.618, -13.193)
**Mental health**	-8.556 (-11.215, -5.896)	-13.516 (-16.67, -10.361)	-11.247 (-14.149, -8.345)	-13.559 (-16.802, -10.315)	-12.275 (-15.433, -9.117)
**Physical component score**	-8.602 (-9.737, -7.468)	-7.207 (-8.663, -5.751)	-9.28 (-10.534, -8.026)	-7.738 (-9.209, -6.266)	-5.23 (-6.716, -3.745)
**Mental component score**	-4.758 (-6.311, -3.205)	-7.623 (-9.462, -5.784)	-5.276 (-6.984, -3.567)	-7.99 (-9.875, -6.106)	-6.78 (-8.627, -4.933)

	Vascular occlusion	Hair loss	Tinnitus	Smell impairment	Impaired sense of taste
**Physical functioning**	-17.739 (-24.204, -11.275)	-7.493 (-11.219, -3.767)	-9.598 (-12.948, -6.249)	-5.012 (-7.966, -2.058)	-6.685 (-10.05, -3.321)
**Role physical**	-33.215 (-44.92, -21.509)	-11.074 (-17.854, -4.294)	-18.601 (-24.695, -12.506)	-8.541 (-13.949, -3.133)	-10.045 (-16.165, -3.926)
**Bodily pain**	-26.837 (-35.921, -17.752)	-10.822 (-16.117, -5.527)	-15.071 (-19.817, -10.326)	n.s.	n.s.
**General health**	-19.066 (-26.063, -12.069)	-7.716 (-11.745, -3.688)	-11.529 (-15.164, -7.894)	-5.031 (-8.238, -1.823)	n.s.
**Vitality**	-13.568 (-21.459, -5.678)	-9.579 (-14.038, -5.12)	-13.799 (-17.805, -9.794)	-5.217 (-8.787, -1.647)	n.s.
**Social functioning**	-10.197 (-19.126, -1.268)	-12.229 (-17.276, -7.181)	-14.019 (-18.622, -9.416)	-5.498 (-9.556, -1.44)	n.s.
**Role emotional**	-17.401 (-30.362, -4.441)	-13.483 (-20.839, -6.127)	-19.79 (-26.454, -13.127)	-7.625 (-13.539, -1.712)	n.s.
**Mental health**	n.s.	-6.967 (-10.758, -3.177)	-11.427 (-14.85, -8.005)	-5.854 (-8.875, -2.832)	-3.463 (-6.924, -0.002)
**Physical component score**	-11.157 (-14.188, -8.126)	-3.408 (-5.19, -1.625)	-4.765 (-6.362, -3.167)	-1.573 (-2.989, -0.157)	-1.836 (-3.445, -0.226)
**Mental component score**	n.s.	-4.536 (-6.737, -2.336)	-6.568 (-8.562, -4.574)	-2.902 (-4.664, -1.139)	n.s.

Supplementary Table 2. Results of uni- and multivariable binary logistic regression modeling to assess the risk of specific persistent symptoms in the occurrence of post-COVID-disease in all participants with sustained complaints.

Variable	Univariable		Multivariable (adjusted for age and sex)	
	**OR (95% CI)**	***p* value**	**Adjusted OR (95% CI)**	***p* value**
Fatigue	10.644 (7.068, 16.031)	<0.001	9.601 (6.35, 14.517)	<0.001
Dizziness	7.768 (5.219, 11.561)	<0.001	6.707 (4.465, 10.074)	<0.001
Memory impairment	7.415 (5.206, 10.561)	<0.001	6.995 (4.871, 10.045)	<0.001
Respiratory problems	6.885 (4.84, 9.796)	<0.001	6.09 (4.244, 8.738)	<0.001
Pain	6.557 (4.55, 9.447)	<0.001	5.789 (3.906, 8.579)	<0.001
Reduced mobility	6.063 (4.252, 8.645)	<0.001	5.813 (3.89, 8.687)	<0.001
Muscular problems	6.907 (4.619, 10.327)	<0.001	6.346 (4.176, 9.644)	<0.001
Sleep disturbance	5.381 (3.771, 7.679)	<0.001	4.784 (3.31, 6.913)	<0.001
Intestinal dysfunction	4.054 (2.739, 6.0)	<0.001	3.749 (2.513, 5.595)	<0.001
Vascular occlusion	4.503 (2.076, 9.768)	<0.001	3.805 (1.724, 8.398)	<0.001
Hair loss	2.891 (1.838, 4.547)	<0.001	2.641 (1.655, 4.215)	<0.001
Tinnitus	4.335 (2.867, 6.554)	<0.001	4.012 (2.613, 6.161)	<0.001
Smell impairment	1.958 (1.33, 2.883)	<0.001	1.989 (1.336, 2.96)	<0.001
Impaired sense of taste	1.677 (1.077, 2.612)	0.026	1.718 (1.09, 2.707)	0.02
Number of persistent symptoms	1.657 (1.54, 1.784)	<0.001	1.643 (1.523, 1.773)	<0.001

Supplementary Table 3. Results of uni- and multivariable binary logistic regression modeling to assess the risk of symptoms within the acute phase of infection for the occurrence of post-COVID-disease in all participants.

Variable	Univariable		Multivariable (adjusted for age and sex)	
	**OR (95% CI)**	***p* value**	**Adjusted OR (95% CI)**	***p* value**
Asymptomatic	0.354 (0.151, 0.832)	0.012	0.322 (0.124, 0.835)	0.02
Dizziness	4.211 (2.996, 5.918)	<0.001	4.011 (2.828, 5.689)	<0.001
Shortness of breath	3.907 (2.794, 5.464)	<0.001	3.639 (2.588, 5.118)	<0.001
Fatigue	3.384 (1.971, 5.81)	<0.001	3.389 (1.957, 5.87)	<0.001
Limb pain	2.171 (1.515, 3.112)	<0.001	2.109 (1.462, 3.043)	<0.001
Nausea	2.971 (1.956, 4.513)	<0.001	2.354 (1.526, 3.63)	<0.001
Diarrhoea	2.247 (1.496, 3.374)	<0.001	2.283 (1.507, 3.46)	<0.001
Vomiting	2.603 (1.15, 5.892)	0.024	2.117 (0.915, 4.895)	0.08
Fever	1.257 (0.91, 1.738)	0.183	1.269 (0.912, 1.766)	0.158
Sore throat	1.451 (1.051, 2.003)	0.026	1.643 (1.162, 2.323)	0.005
Loss of smell/taste	1.106 (0.801, 1.528)	0.566	1.188 (0.851, 1.659)	0.312
Cough	1.708 (1.224, 2.385)	0.002	1.824 (1.293, 2.574)	<0.001
Number of acute symptoms	1.358 (1.258, 1.466)	<0.001	1.361 (1.257, 1.474)	<0.001

Supplementary Table 4. Results of uni- and multivariable binary logistic regression modeling to assess risk factors for the occurrence of post-COVID-disease in the total cohort (n=909).

Variable	Univariable		Multivariable (adjusted for age and sex)	
	OR (95% CI)	p value	Adjusted OR (95% CI)	p value
Age	1.021 (1.011, 1.031)	<0.001		
Sex, favoring female	1.863 (1.316, 2.639)	<0.001		
No pre-existing conditions	0.412 (0.295, 0.576)	<0.001	0.464 (0.322, 0.669)	<0.001
Hypertension	2.007 (1.413, 2.85)	<0.001	1.727 (1.148, 2.598)	0.009
Diabetes mellitus	4.359 (2.372, 8.012)	<0.001	3.93 (2.044, 7.556)	<0.001
Kidney disease	1.522 (0.472, 4.907)	0.507	1.18 (0.356, 3.906)	0.787
Cancer	3.028 (1.443, 6.353)	0.005	2.514 (1.16, 5.446)	0.019
Liver disase	3.093 (1.509, 6.342)	0.003	2.986 (1.422, 6.271)	0.004
Lung disease	3.117 (1.993, 4.875)	<0.001	2.951 (1.868, 4.661)	<0.001
Obesity	2.117 (1.446, 3.1)	<0.001	1.956 (1.319, 2.899)	<0.001
Active smoking	1.462 (0.946, 2.259)	0.095	1.701 (1.086, 2.666)	0.02
Active and prior smoking	1.525 (1.061, 2.191)	0.025	1.691 (1.161, 2.465)	0.006
Any permanent medication	3.396 (2.409, 4.787)	<0.001	2.942 (2.016, 4.292)	<0.001
Polypharmacy (≥5 drugs daily)	6.273 (3.309, 11.89)	<0.001	5.036 (2.506, 10.121)	<0.001
Time after infection (categorical)	1.006 (0.865, 1.171)	0.933	0.999 (0.855, 1.168)	0.993
Time after infection (days)	0.999 (0.998, 1.001)	0.253	0.999 (0.997, 1.001)	0.228
WHO	1.548 (1.216, 1.971)	<0.001	1.539 (1.198, 1.976)	<0.001
In-patient	1.022 (0.437, 2.392)	0.96	1.012 (0.423, 2.421)	0.978
oxygen treatment	0.4 (0.045, 3.527)	0.57	0.291 (0.017, 4.909)	0.392
In patient (ICU admission)	0.542 (0.048, 6.144)	0.617	0.138 (0.003, 5.951)	0.302
Vaccination before	1.048 (0.614, 1.789)	0.89	0.971 (0.562, 1.676)	0.915

Legend of the supplementary figures

Supplementary Figure 1. Within-cluster sum of square (WCSS) depending on the number of clusters in k-means analysis of the cohort with persistent symptoms (n=643).

Supplementary Figure 2. Number of symptoms of acute SARS-CoV-2 infection and persistent symptoms in a population-based cohort of SARS-CoV-2 survivors (maximum of acute phase=10, prolonged and persistent phase=14).

Supplementary Figure 3. Time-dependent distribution of participants. A. Median time interval (days) between acute infection and the survey. Time-dependent representation of the frequency of participants with persistent complaints (B) and the identified post-COVID clusters (C) related to the total cohort.

Supplementary Figure 4. Comparison of the monthly incidence in Jena with the number of survey participants infected with SARS-CoV-2 in the given month.

Supplementary Figure 5. Frequency of persistent post-COVID associated symptoms in a population-based cohort of SARS CoV-2 survivors. The proportions of patients with each symptom are shown.

Supplementary Figure 6. Analysis of the individual properties integrated in the cluster analysis (SF-36 dimensions and sum scales), plotted on a normalized scale (z-score) (* $p<0.001$).

Supplementary Figure 7. Relative frequency of satisfaction with personal health (A) and self-reported QoL (B) on a Likert scale in SARS-CoV-2 survivors, according to the occurrence of persistent symptoms and the influence of SF-36 results. The proportions of participants are shown as diverging stacked bar charts (* $p<0.001$ Kruskal-Wallis test).

Supplementary Figure 8. Frequency of acute symptoms in a population-based cohort of SARS CoV-2 survivors, according to persistent symptoms. The proportions of participants with each symptom as well as the proportion of asymptomatic individuals are shown (* $p\leq0.05$; ** $p\leq0.01$; *** $p\leq0.001$).

Supplementary Figure 9. Venn diagram of identified fatigue and depression in the post-COVID cohort and specified post-COVID disease cluster, according to FAS and PHQ-9. Overall, 307 people (48.6%) with persistent symptoms, and 169 (91.4%) and 138 (30.9%) in the identified post-COVID subclusters, respectively, experienced both fatigue and depression. The proportion of co-occurrence of having both, fatigue and depression was significantly higher in post-COVID disease cluster 1.

Supplementary Figure 1. Within-cluster sum of square (WCSS) depending on the number of clusters in k-means analysis of the cohort with persistent symptoms (n=643).

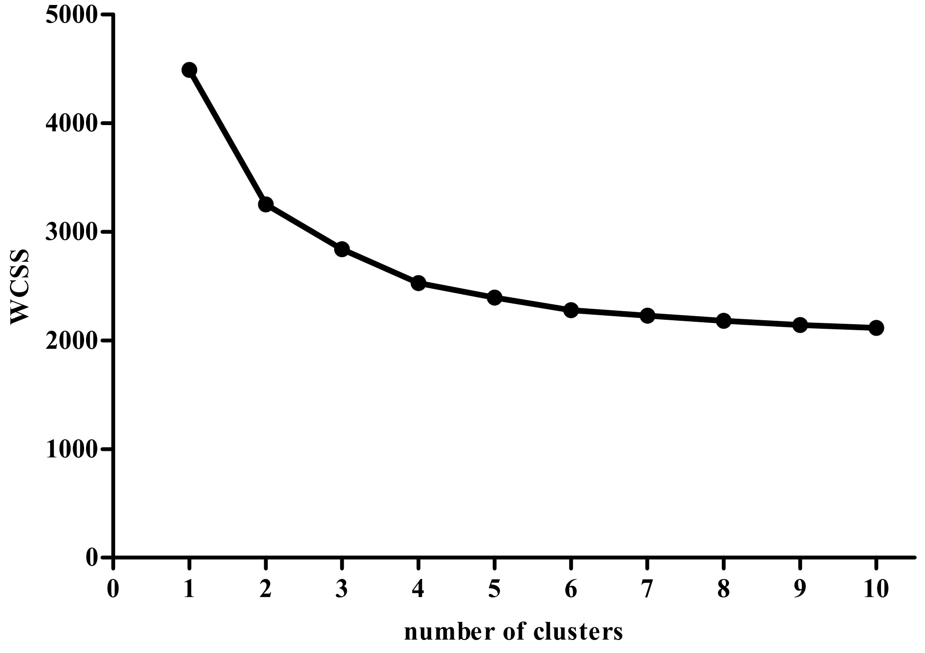

Supplementary Figure 2. Number of symptoms of acute SARS-CoV-2 infection and persistent symptoms in a population-based cohort of SARS-CoV-2 survivors (maximum of acute phase=10, prolonged and persistent phase=14).

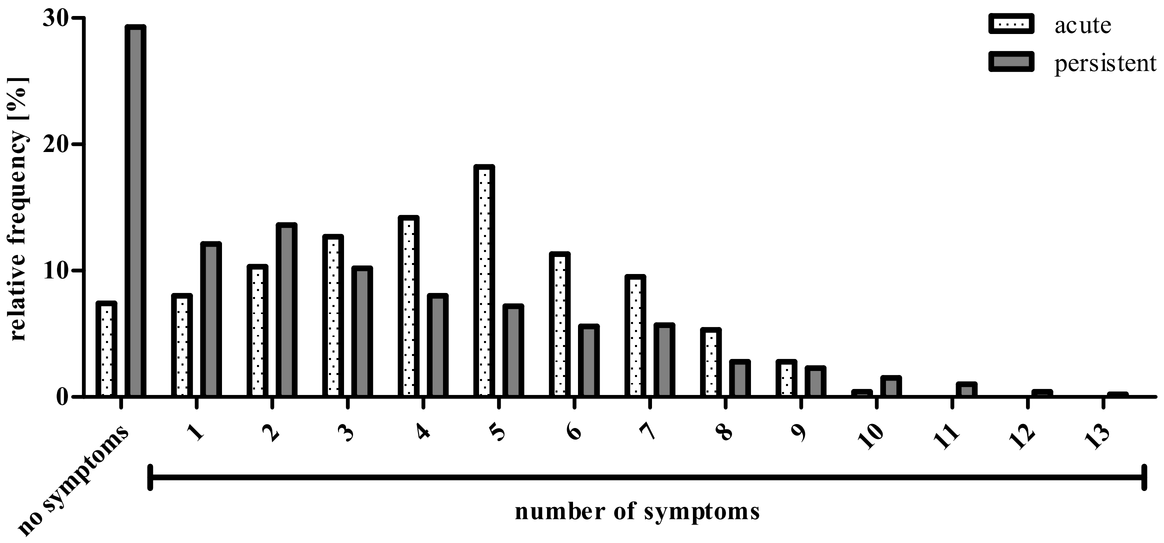

Supplementary Figure 3. Time-dependent distribution of participants. A. Median time interval (days) between acute infection and the survey. Time-dependent representation of the frequency of participants with persistent complaints (B) and the identified post-COVID clusters (C) related to the total cohort.

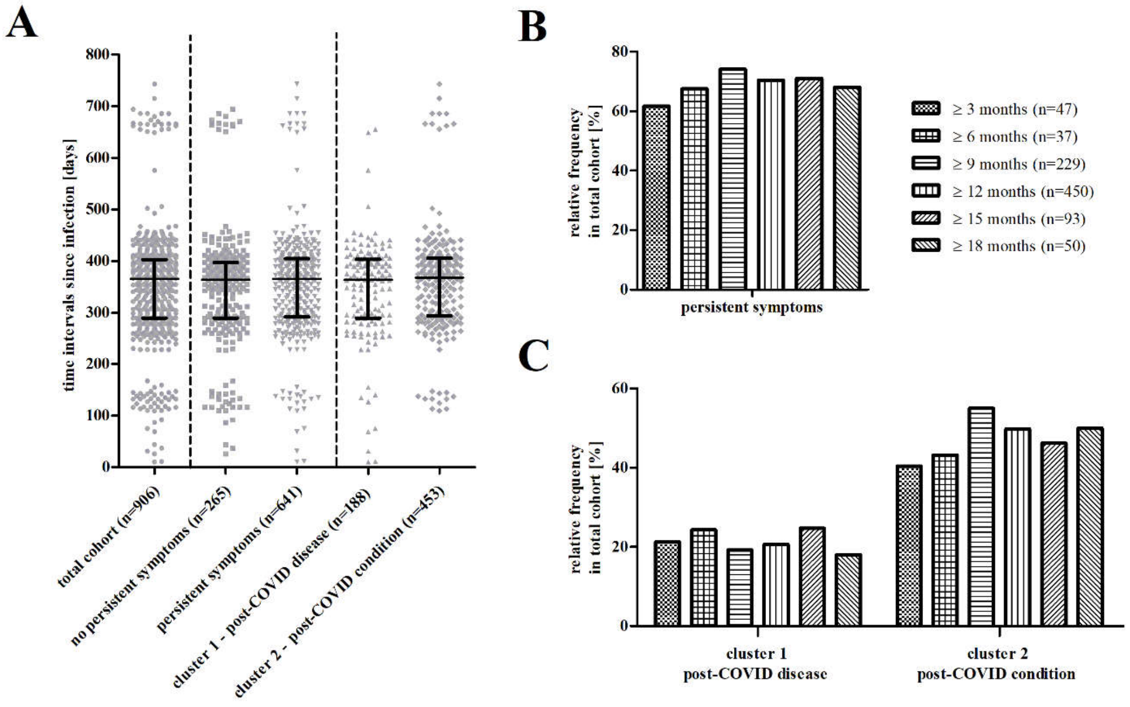

Supplementary Figure 4. Comparison of the monthly incidence in Jena with the number of survey participants infected with SARS-CoV-2 in the given month.

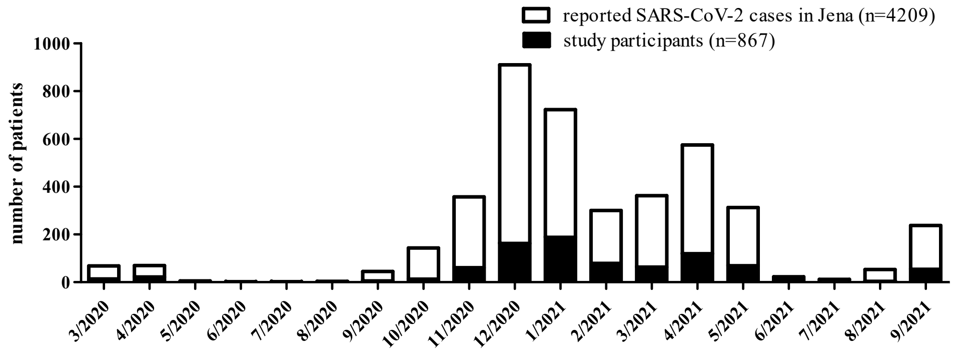

reported SARS-CoV-2 cases in Jena	69	70	5	2	2	4	45	144	358	910	723	301	362	574	313	23	12	54
study participants	14	22	0	0	1	0	5	13	61	162	188	79	63	119	69	13	0	4
percentage	20,3	31,4	0	0	50	0	11,1	9	17	17,8	26	26,2	17,4	20,7	22	56,5	0	7,4

Supplementary Figure 5. Frequency of persistent post-COVID associated symptoms in a population-based cohort of SARS CoV-2 survivors. The proportions of patients with each symptom are shown.

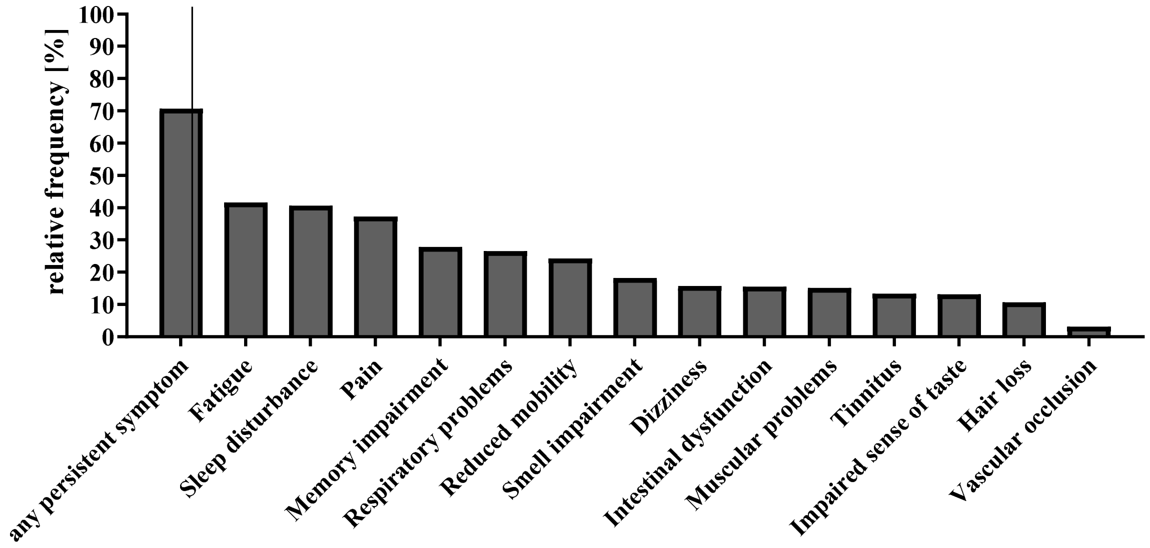

Supplementary Figure 6. Analysis of the individual properties integrated in the cluster analysis (SF-36 dimensions and sum scales), plotted on a normalized scale (z-score) (* p<0.001).

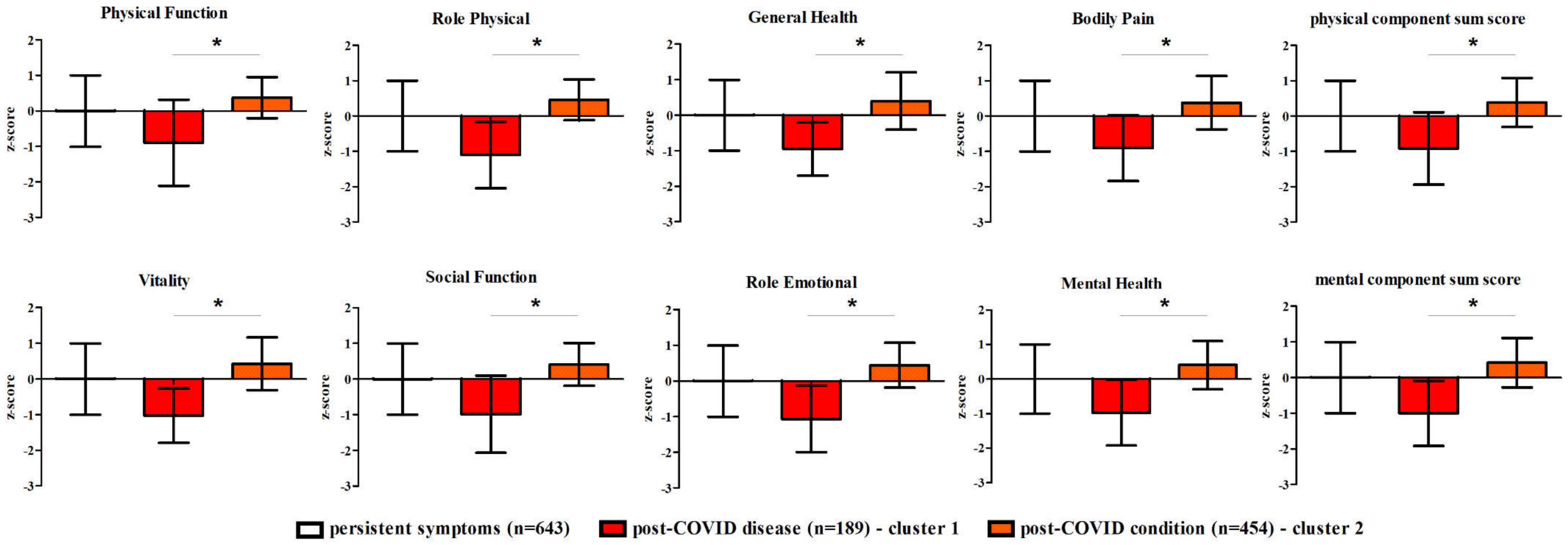

Supplementary Figure 7. Relative frequency of satisfaction with personal health (A) and self-reported QoL (B) on a Likert scale in SARS-CoV-2 survivors, according to the occurrence of persistent symptoms and the influence of SF-36 results. The proportions of participants are shown as diverging stacked bar charts (* p<0.001 Kruskal-Wallis test).

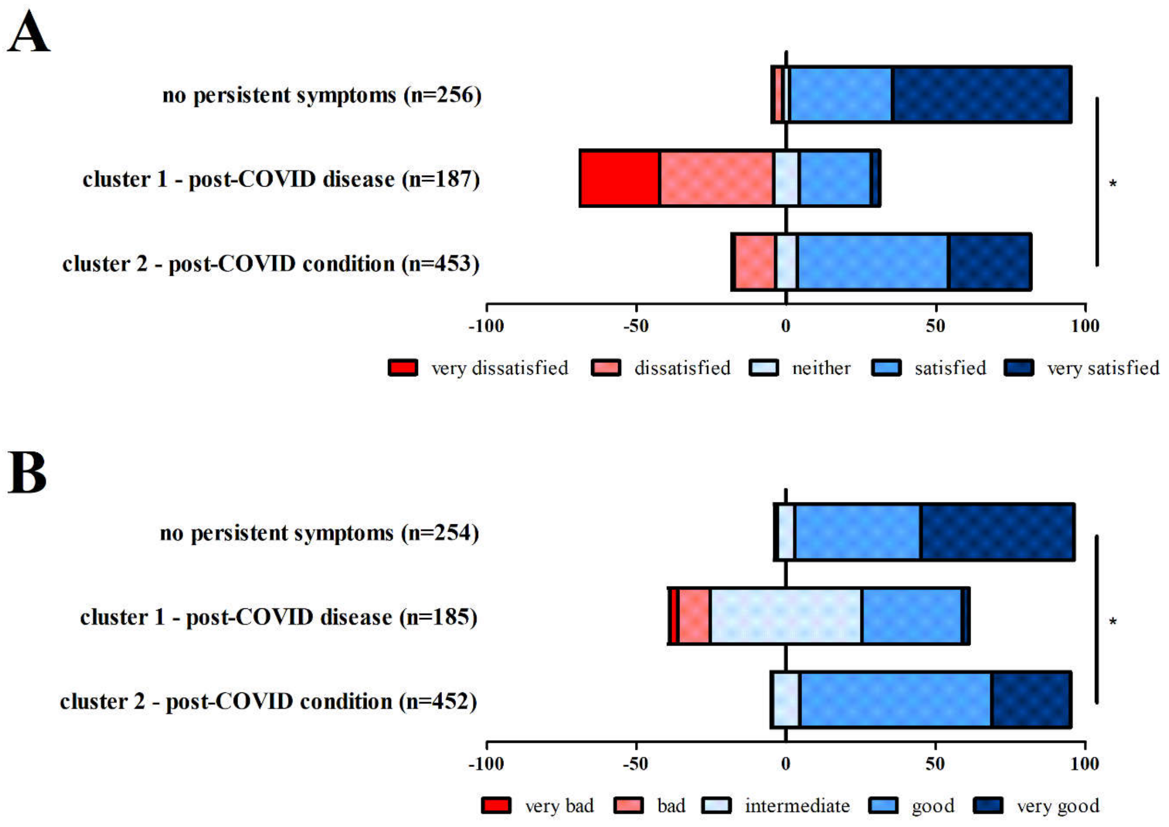

Supplementary Figure 8. Frequency of acute symptoms in a population-based cohort of SARS CoV-2 survivors, according to persistent symptoms. The proportions of participants with each symptom as well as the proportion of asymptomatic individuals are shown (* p≤0.05; ** p≤0.01; *** p≤0.001).

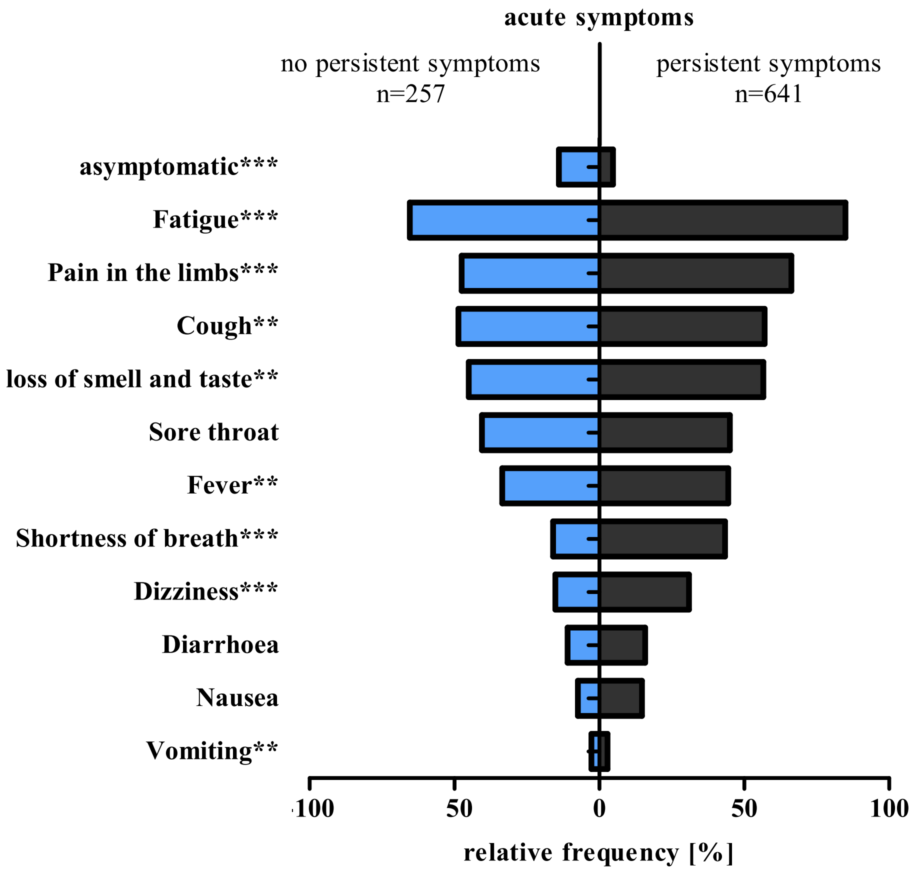

Supplementary Figure 9. Venn diagram of identified fatigue and depression in the post-COVID cohort and specified post-COVID disease cluster, according to FAS and PHQ-9. Overall, 307 people (48.6%) with persistent symptoms, and 169 (91.4%) and 138 (30.9%) in the identified post-COVID subclusters, respectively, experienced both fatigue and depression. The proportion of co-occurrence of having both, fatigue and depression was significantly higher in post-COVID disease cluster 1.

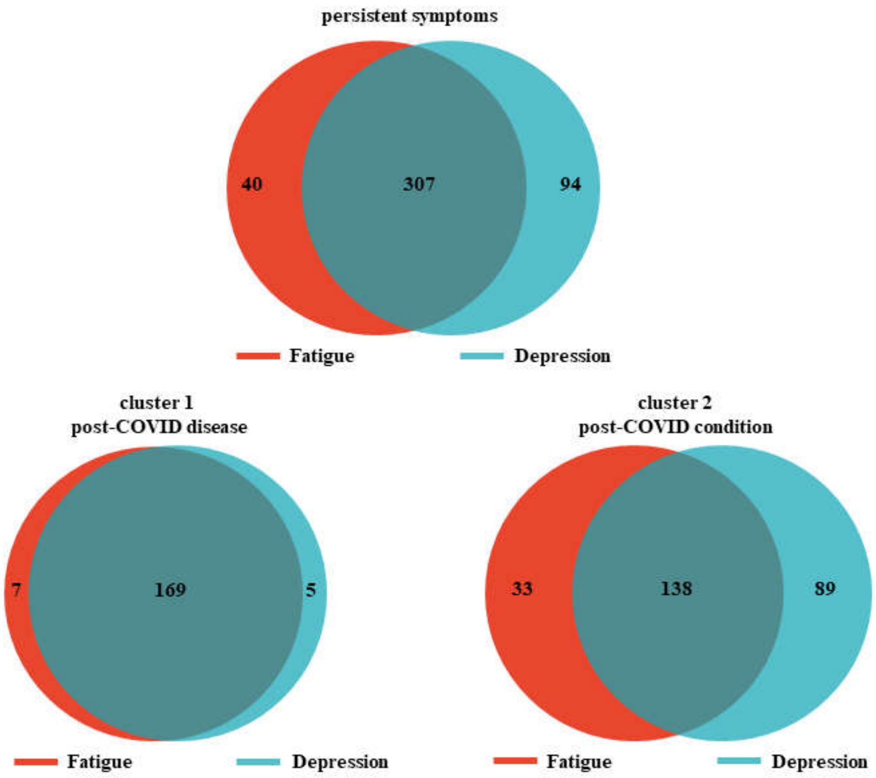

Supplement: Supplementary file 1 — Supplementary file1 (PDF 628 kb) [file 15010_2022_1886_MOESM1_ESM.pdf]
